# Monitoring of Anticoagulant Activity of Dabigatran and Rivaroxaban in the Presence of Heparins

**DOI:** 10.3390/jcm11082236

**Published:** 2022-04-16

**Authors:** Aleksandra Jakimczuk, Bartlomiej Kalaska, Kamil Kamiński, Joanna Miklosz, Shin-Ichi Yusa, Dariusz Pawlak, Krzysztof Szczubiałka, Andrzej Mogielnicki

**Affiliations:** 1 Department of Pharmacodynamics, Medical University of Bialystok, 15-089 Bialystok, Poland; aleksandra.jakimczuk@sd.umb.edu.pl (A.J.); joanmiklosz@gmail.com (J.M.); dariusz.pawlak@umb.edu.pl (D.P.); andrzej.mogielnicki@umb.edu.pl (A.M.); 2 Faculty of Chemistry, Jagiellonian University, Gronostajowa 2, 30-387 Krakow, Poland; krzysztof.szczubialka@uj.edu.pl; 3 Department of Applied Chemistry, Graduate School of Engineering, University of Hyogo, Himeji 671-2280, Japan; yusa@eng.u-hyogo.ac.jp

**Keywords:** anticoagulants, blood coagulation tests, dabigatran, heparin, laboratory monitoring, rivaroxaban

## Abstract

The routine monitoring of direct oral anticoagulants (DOACs) may be considered in patients with renal impairment, patients who are heavily obese, or patients requiring elective surgery. Using the heparin-binding copolymer (HBC) and polybrene, we aimed to develop a solution for monitoring the anticoagulant activity of DOACs in human plasma in the interfering presence of unfractionated heparin (UFH) and enoxaparin. The thrombin time (TT) and anti-factor Xa activity were monitored in pooled plasma from healthy volunteers. In these tests, plasma with dabigatran or rivaroxaban was mixed with UFH or enoxaparin and then incubated with HBC or polybrene, respectively. HBC and polybrene neutralized heparins and enabled monitoring of anticoagulant activity of dabigatran in the TT test. Both agents allowed for accurate measurement of anti-factor Xa activity in the plasma containing rivaroxaban and heparins in the concentration range reached in patients’ blood. Here, we present diagnostic tools that may improve the control of anticoagulation by eliminating the contamination of blood samples with heparins and enabling the monitoring of DOACs’ activity.

## 1. Introduction

Unfractionated heparin (UFH) or low-molecular-weight heparins (LMWHs) are parenteral anticoagulants used in the prophylaxis and treatment of patients with thrombotic disorders. Heparins are also administered to prevent blood clotting during hemodialysis or surgical procedures involving extracorporeal circulation or central venous catheters (CVCs). Because of a more convenient route of their administration and a more predictable pharmacokinetic profile, direct oral anticoagulants (DOACs) have become an attractive alternative to parenteral anticoagulants. In contrast to vitamin K antagonists, DOACs do not usually require dose adjustment based on the results of laboratory tests, although the measurement of their anticoagulant activity may be needed in patients with renal impairment, patients who are heavily obese, when switching from one anticoagulant to another, and to assess compliance or requiring elective surgery [1,2,3]. Thrombin time (TT) or diluted TT and ecarin chromogenic assay are quantitative tests for direct thrombin inhibitors, such as dabigatran, and the anti-factor Xa assay is recommended for direct anti-factor Xa inhibitors, such as rivaroxaban. A problem with measuring DOACs’ activity may arise if blood samples also contain heparins, because of the synergistic action of both anticoagulants [4]. Contamination with heparins may occur in patients requiring CVC connection; for example, those with renal failure, cancer, or malnutrition [5,6]. Although concomitant administration of heparin and DOAC is rare in clinical practice, in some situations (e.g., patients on DOAC treatment who receive LMWH before/after surgery or during hemodialysis), LMWHs may remain in the blood and disturb the precise measurement of DOACs’ activity [4,7,8,9,10].

To enable monitoring of anticoagulant activity of different anticoagulant drugs or hemostasis pathology in the presence of heparins, heparin inhibitors such as protamine, heparinase, and polybrene were used in several clotting assays [4,7,9,11,12,13,14,15]. However, there are still issues limiting their usefulness. Protamine may interact with clotting [16,17,18], which makes it unsuitable for in vitro neutralization tests. A heparinase requires a lengthy preparation of a plasma sample [7,12]. Both protamine and heparinase are proteins that can be unstable at room temperature or contain source-related impurities. A synthetic and stable macromolecular compound—polybrene (hexadimethrine bromide)—was most extensively tested for inhibition of the residual heparins in vitro [12,13,14,15,19], and seems to be the most promising. The BIOPHEN™ DiXaI kit of anti-factor Xa chromogenic assay with a heparin inhibitor was designed to resolve the issue of heparin contamination during monitoring of direct factor Xa inhibitors’ activity [20]. However, polybrene/DiXaI have some drawbacks, e.g., they may slightly change the basal values of the coagulation test depending on the concentration used, or they cannot neutralize large amounts of heparins [9,15,21]. Moreover, there is still no evidence of efficient heparin neutralization in other tests and anticoagulants, such as dabigatran.

Recently, we have developed a heparin-binding copolymer (HBC) composed of a neutral poly(ethylene glycol) (PEG) block and a cationic poly(3-(methacryloylamino) propyl trimethylammonium chloride) (PMAPTAC) block. The mechanism of HBC action involves electrostatic binding between cationic groups of HBC and anionic groups of heparin-based anticoagulants. We have proven its high efficacy in reversing the effects of heparins and heparin mimetics in vitro in human plasma and in vivo in rats and mice [22,23,24,25].

The International Council for Standardization in Haematology guidelines recommended measuring heparin independently of DOACs while heparin bridging in DOAC-treated patients [26,27]. In the present study, we propose a reverse approach to measure DOACs separate from heparin interference. Therefore, we have investigated whether the use of HBC or polybrene would allow monitoring of the anticoagulant activity of dabigatran by TT test and rivaroxaban by anti-factor Xa activity in human plasma containing heparins.

## 2. Materials and Methods

### 2.1. Materials

We used polybrene (Sigma-Aldrich, Taufkirchen, Germany), UFH (Polfa, Warszawa, Poland), enoxaparin (Sanofi-Aventis, Paris, France), dabigatran and rivaroxaban plasma calibrators (BIOPHEN, Hyphen-Biomed, France), phosphate-buffered saline (PBS) (Biomed, Lublin, Poland), TT reagents (Bio-Ksel, Grudziądz, Poland), and anti-factor Xa assay kits (BioMedica Diagnostics, Windsor, NS, Canada). HBC was synthesized as previously described [22].

### 2.2. Plasma Samples

Blood samples were collected from eight healthy volunteers into trisodium citrate (9:1 *v*/*v*) vacuettes (Vacuette, Greiner Bio-One GmbH, Kremsmünster, Austria). The group consisted of four women and four men between the ages of 25 and 45 years, with a negative history of bleeding and thrombosis, with clotting times in the normal range (activated partial thromboplastin time (aPTT) = 33.8 ± 1.7 s (s), prothrombin time (PT) = 10.4 ± 0.2 s, international normalized ratio (INR) = 1.2 ± 0.1, and TT = 14.3 ± 1.4 s), and without taking any medications at the time of sample collection. The demographic and clinical characteristics of the healthy volunteers are summarized in Table S1 in the Supplementary Materials. The procedure was approved by the Local Ethics Committee of the Medical University of Bialystok (Permit No. R-I-002/193/2019). Plasma samples were obtained by centrifugation at 3500× *g* for 20 min at 4 °C. The final concentrations of DOACs (50, 100, 150, 200, and 250 ng/mL) were derived by diluting calibrators in normal human pooled plasma. Heparins, HBC, and polybrene solutions were diluted in saline solution accordingly to obtain final concentrations: 0.25, 0.5, 1.0, 1.5, and 2.0 U/mL for UFH; 2.5, 5, 10, 15, and 20 µg/mL for enoxaparin; and 10, 25, 50, and 100 µg/mL for HBC and polybrene. Pooled plasma spiked with PBS served as a control group.

### 2.3. TT Test

TT was measured using a coagulometer Coag Chrom 4000 (Bio-ksel, Poland). The TT reagent contained bovine thrombin at a concentration of 3.0 UNIH/mL. Samples without DOAC were prepared by mixing pooled plasma (200 µL) with increasing concentrations of UFH (0.25, 0.5, 1.0, 1.5, and 2.0 U/mL) or enoxaparin (2.5, 5, 10, 15, and 20 µg/mL) and HBC or polybrene (10 µL of 50 µg/mL). Plasmas with dabigatran (50 ng/mL) were divided into three groups: (1) dabigatran alone, (2) mixed with UFH or enoxaparin, and (3) mixed with UFH or enoxaparin and HBC or polybrene in chosen concentrations. After 2 min of incubation (37 °C), thrombin (100 µL) was added to the mixture (100 µL) and TT was measured immediately. If TT was more than or equal to 300.0 s, a value of 300.0 s was ascribed for the sake of statistical analysis. The results from the TT test were presented in seconds and, additionally, converted to ng/mL using a dabigatran calibrator.

### 2.4. Anti-Factor Xa Activity Assay

The anti-factor Xa activity was measured in a 96-well plate reader (Synergy HTX, BioTek, Winooski, VT, USA) according to the modified kit manufacturer’s instructions. In brief, a solution (10 µL) of UFH (0.25, 0.5, 1.0, 1.5, and 2.0 U/mL) or enoxaparin (2.5, 5, 10, 15, and 20 µg/mL) and HBC (10 µg/mL) or polybrene (50 µg/mL) was added to pooled plasma (200 µL). Then, an antithrombin III reagent (80 µL) was added to the previously prepared mixture (10 µL) and incubated at 37 °C for 2 min. Then, bovine factor Xa reagent (80 µL) was added, mixed, and incubated at 37 °C for 1 min. After incubation, spectrozyme factor Xa (80 µL) was added, mixed, and incubated at 37 °C for 5 min, and the anti-factor Xa activity was measured at 405 nm. Plasmas with rivaroxaban (50 ng/mL) were divided into three groups: (1) rivaroxaban alone, (2) mixed with UFH or enoxaparin, and (3) mixed with UFH or enoxaparin and HBC or polybrene in chosen concentrations, and the anti-factor Xa activity was measured as described previously. The results from the anti-factor Xa assay were presented as anti-factor Xa activity based on the UFH calibration curve (U/mL) and, additionally, converted to ng/mL using a rivaroxaban calibrator.

### 2.5. Statistical Analysis

Shapiro–Wilk’s test was used for data distribution analysis. The data were presented as violin plots with minimum and maximum values and analyzed using the non-parametric Mann-Whitney test. A two-tailed *p* < 0.05 was considered statistically significant. The data were analyzed using GraphPad Prism 8 (GraphPad Software, San Diego, CA, USA).

## 3. Results

### 3.1. The Effect of Anticoagulants, HBC, and Polybrene on the TT Test in Human Plasma

**Figure 1:**
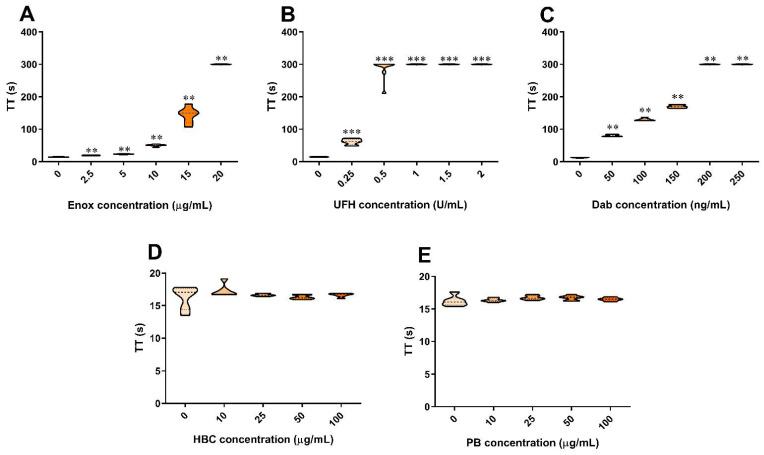
Effect of enoxaparin (**A**), unfractionated heparin (**B**), dabigatran (**C**), HBC (**D**), and polybrene (**E**) on TT in human plasma. ** *p* < 0.01, *** *p* < 0.001 vs. control, Mann–Whitney test. The results are shown as violin plots with minimum and maximum values and analyzed with GraphPad Prism 8 software. Dab, dabigatran; Enox, enoxaparin; HBC, heparin-binding copolymer; PB, polybrene; TT, thrombin time; UFH, unfractionated heparin.

**Figure 2:**
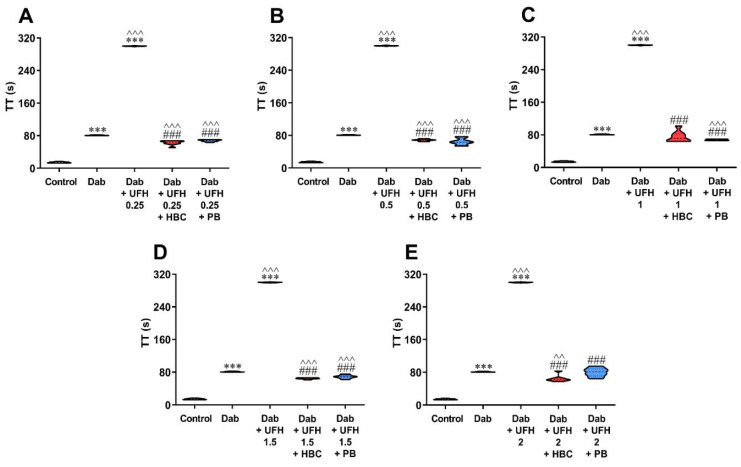
Inhibition of UFH at a concentration of 0.25 (**A**), 0.5 (**B**), 1 (**C**), 1.5 (**D**), and 2 U/mL (**E**) by HBC or polybrene at a concentration of 50 µg/mL during testing of dabigatran (50 ng/mL) anticoagulant activity by TT in human plasma. *** *p* < 0.001 vs. control; ^^ *p* < 0.01, ^^^ *p* < 0.001 vs. dabigatran; ### *p* < 0.001 vs. dabigatran with UFH, Mann–Whitney test. The results are shown as violin plots with minimum and maximum values and analyzed with GraphPad Prism 8 software. Dab, dabigatran; HBC, heparin-binding copolymer; PB, polybrene; TT, thrombin time; UFH; unfractionated heparin.

Enoxaparin, UFH, and dabigatran prolonged TT in a concentration-dependent manner (Figure 1A–C). Neither HBC nor polybrene affected TT (Figure 1D,E). The addition of UFH and enoxaparin at all concentration ranges to plasma containing dabigatran significantly prolonged TT when compared with dabigatran alone (Figure 2A–E).

### 3.2. Inhibition of UFH by HBC or Polybrene in the Presence of Dabigatran in the TT Test Measured in Human Plasma

Both HBC and polybrene at a concentration of 50 µg/mL neutralized the anticoagulant activity of UFH and lowered TT to levels achieved in plasma samples containing only dabigatran. We observed lower values of TT (by 15–23%) after adding HBC or polybrene to plasma with UFH and dabigatran in comparison with dabigatran alone (Figure 2A–E). We observed similar results when the concentration was converted to ng/mL using a dabigatran calibrator (Figure S1, Supplementary Materials).

### 3.3. Inhibition of Enoxaparin by HBC or Polybrene in the Presence of Dabigatran in the TT Test Measured in Human Plasma

**Figure 3:**
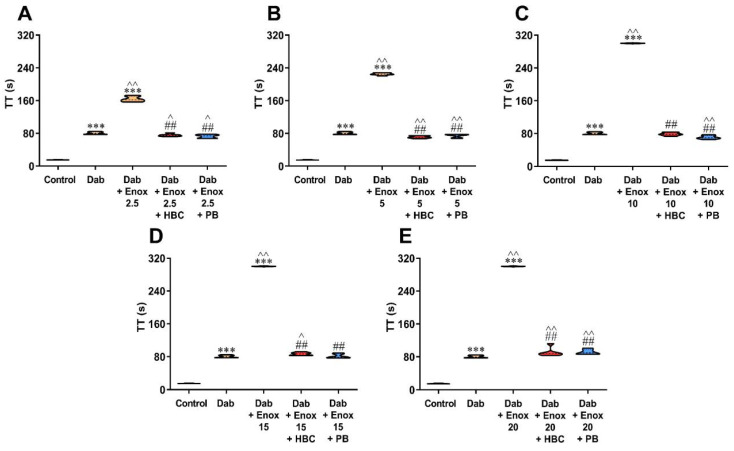
Inhibition of enoxaparin at a concentration of 2.5 (**A**), 5 (**B**), 10 (**C**), 15 (**D**), and 20 µg/mL (**E**) by HBC or polybrene at a concentration of 50 µg/mL during testing of dabigatran (50 ng/mL) anticoagulant activity by TT in human plasma. *** *p* < 0.001 vs. control; ^ *p* < 0.05, ^^ *p* < 0.01 vs. dabigatran; ## *p* < 0.01 vs. dabigatran with enoxaparin, Mann–Whitney test. The results are shown as violin plots with minimum and maximum values and analyzed with GraphPad Prism 8 software. Dab, dabigatran; Enox, enoxaparin; HBC, heparin-binding copolymer; PB, polybrene; TT, thrombin time.

Both HBC and polybrene at a concentration of 50 µg/mL restored TT prolonged by enoxaparin to levels achieved by dabigatran itself. Compared with dabigatran alone, we found lower (6–12%) values of TT in the presence of HBC and polybrene if enoxaparin was added at a concentration up to 10 µg/mL (Figure 3A–C), and higher (8–14%) if enoxaparin was added at a concentration of 15 µg/mL and above (Figure 3D,E). We observed similar results when the concentration was converted to ng/mL using a dabigatran calibrator (Figure S2, Supplementary Materials).

### 3.4. The Effect of Anticoagulants, HBC, and Polybrene on the Anti-Factor Xa Assay in Human Plasma

**Figure 4:**
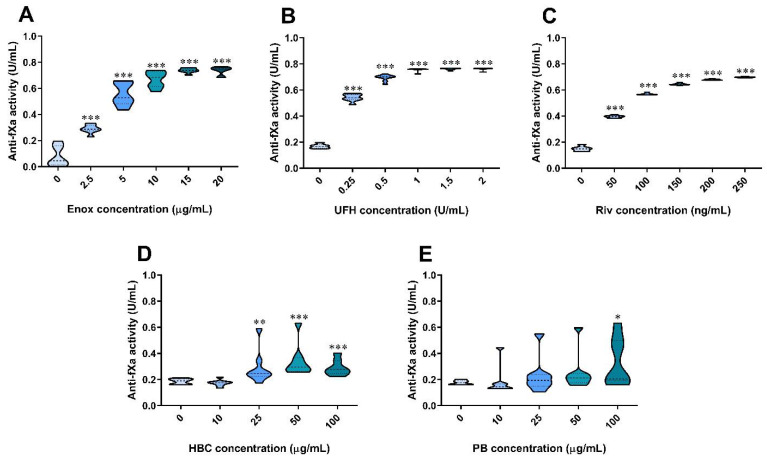
Effect of enoxaparin (**A**), UFH (**B**), rivaroxaban (**C**), HBC (**D**), and polybrene (**E**) on anti-factor Xa (anti-fXa) assay in human plasma. * *p* < 0.05, ** *p* < 0.01, *** *p* < 0.001 vs. control, Mann–Whitney test. The results are shown as violin plots with minimum and maximum values and analyzed with GraphPad Prism 8 software. Enox, enoxaparin; HBC, heparin-binding copolymer; PB, polybrene; Riv, rivaroxaban; UFH; unfractionated heparin.

**Figure 5:**
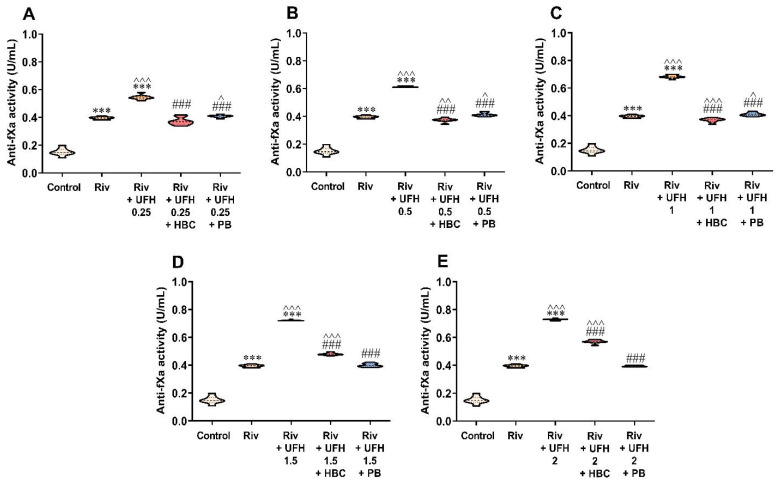
Inhibition of UFH at a concentration of 0.25 (**A**), 0.5 (**B**), 1 (**C**), 1.5 (**D**), and 2 U/mL (**E**) by HBC (10 µg/mL) and polybrene (50 µg/mL) during testing of rivaroxaban (50 ng/mL) anticoagulant activity by anti-factor Xa (anti-fXa) activity in human plasma. *** *p* < 0.001 vs. control; ^ *p* < 0.05, ^^ *p* < 0.01, ^^^ *p* < 0.001 vs. rivaroxaban; ### *p* < 0.001 vs. rivaroxaban with UFH, Mann–Whitney test. The results are shown as violin plots with minimum and maximum values and analyzed with GraphPad Prism 8 software. HBC, heparin-binding copolymer; PB, polybrene; Riv, rivaroxaban; UFH, unfractionated heparin.

The enoxaparin, UFH, and rivaroxaban increased anti-factor Xa activity in a concentration-dependent manner (Figure 4A–C). HBC increased anti-factor Xa activity from the concentration of 25 µg/mL and above, while polybrene did so only at a concentration of 100 µg/mL (Figure 4D,E). Therefore, for neutralization experiments, we chose HBC and polybrene at neutral concentrations of 10 and 50 µg/mL, respectively. Similarly to TT, the addition of UFH and enoxaparin at all concentration ranges to plasma containing rivaroxaban significantly increased anti-factor Xa activity when compared with rivaroxaban alone (Figure 5A–E).

### 3.5. Inhibition of UFH by HBC or Polybrene in the Presence of Rivaroxaban in the Anti-Factor Xa Activity Test Measured in Human Plasma

HBC bound UFH completely up to the concentration of 1 U/mL, with slight (5–6%) differences between groups: rivaroxaban + UFH + HBC versus rivaroxaban alone (Figure 5A–C), and only partially at concentrations of 1.5–2 U/mL, with differences around 22–44% (Figure 5D,E). Polybrene neutralized UFH at all concentration ranges. We found similar results when the concentration was converted to ng/mL using a rivaroxaban calibrator (Figure S3, Supplementary Materials).

### 3.6. Inhibition of Enoxaparin by HBC or Polybrene in the Presence of Rivaroxaban in the Anti-Factor Xa Activity Test Measured in Human Plasma

**Figure 6:**
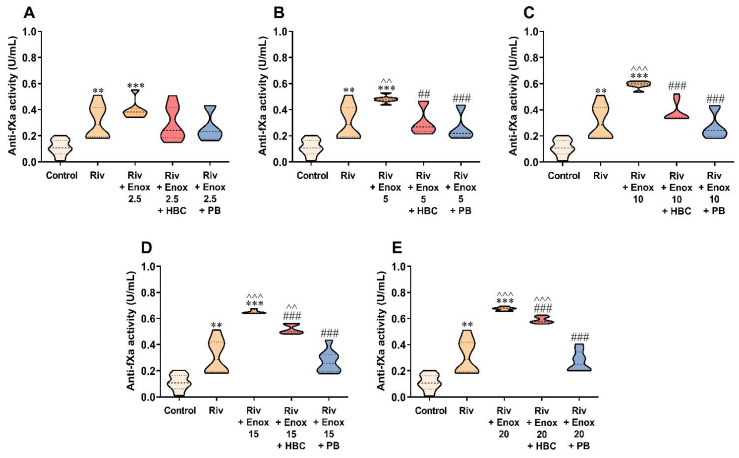
Inhibition of enoxaparin at a concentration of 2.5 (**A**), 5 (**B**), 10 (**C**), 15 (**D**), and 20 µg/mL (**E**) by HBC (10 µg/mL) and polybrene (50 µg/mL) during testing of rivaroxaban (50 ng/mL) anticoagulant activity by anti-factor Xa (anti-fXa) activity in human plasma. ** *p* < 0.01, *** *p* < 0.001 vs. control; ^^ *p* < 0.01, ^^^ *p* < 0.001 vs. rivaroxaban; ## *p* < 0.01, ### *p* < 0.001 vs. rivaroxaban with enoxaparin, Mann–Whitney test. The results are shown as violin plots with minimum and maximum values and analyzed with GraphPad Prism 8 software. Enox, enoxaparin; HBC, heparin-binding copolymer; PB, polybrene; Riv, rivaroxaban.

Similarly to the experiment with UFH, HBC effectively neutralized enoxaparin up to the concentration of 10 µg/mL, and polybrene at the full enoxaparin concentration range, with slight differences when compared with samples with rivaroxaban alone (Figure 6A–E). We observed similar results when the concentration was converted to ng/mL using a rivaroxaban calibrator (Figure S4, Supplementary Materials).

## 4. Discussion

The DOACs’ levels or their anticoagulant effect should be measured in particular conditions; for example, in patients at risk of bleeding, overdosed, or requiring invasive procedures [1,28]. TT is a first-line test used for screening the presence or absence of dabigatran [1,29]. As the anti-factor Xa assay is recommended for the monitoring of anticoagulant activity of both heparins and rivaroxaban, this test is exceptionally prone to error whenever both of these drugs are administered together [4]. In previous studies, heparinase or polybrene showed good heparin-neutralizing activity, although they still interfered with the results of coagulation tests themselves [9,15,21]. In our assay, we selected the neutral concentrations of polybrene and our copolymer, HBC, which would not affect the result of the coagulation tests. We chose the concentrations of DOACs based on our dose–response curves and other studies [30,31].

Blood collection should be performed via peripheral veins. In the case of the unavailability of peripheral veins, patients being in critical condition, and pain during repeated blood sampling [32], the blood can be also collected from a CVC. It relies on flushing 5 mL of 0.9% sodium chloride, and discarding 5 mL of blood, or six times that of the catheter dead space volume, before specimen collection. UFH solution is also used to prevent catheter occlusion and infection, but may contaminate the collected blood samples [5,33]. In this and similar situations, it is impossible to correctly assess the anticoagulation of a DOAC alone. Therefore, a means of selective inactivation of heparins without affecting the anticoagulative effect of DOACs is desired. Polybrene and HBC restored TT increased by UFH to values similar to those observed in the samples with dabigatran alone. Besides, other studies showed heparin inhibitory activity of polybrene in PT, APTT [12,19], activated clotting time [14], and thrombin generation [13,15] assays, but none of them involved the interference of heparin neutralization by polybrene in the presence of dabigatran. Here, we present evidence that polybrene, as well as HBC, may be used to neutralize contaminating UFH in the TT test, allowing for accurate monitoring of dabigatran activity. We noticed a slight shortening of TT by both examined agents in comparison with samples with dabigatran alone, which requires further experimental explanation. We replicated our results using a dabigatran or rivaroxaban calibrator in the TT test and anti-factor Xa assay, respectively, to make their presentation closer to the clinical practice.

Currently, there is evidence that monitoring of rivaroxaban in the presence of UFH is possible [34], although no validated universal heparin inhibitor was developed. We found that polybrene restored anti-factor Xa activity increased by rivaroxaban and UFH to values similar to those observed in the samples with rivaroxaban alone. Additionally, HBC showed activity comparable to polybrene UFH-neutralizing activity up to a UFH concentration of 1 U/mL. Thus, we present tools for accurate monitoring of the anticoagulant activity of dabigatran or rivaroxaban in the presence of UFH regardless of the method of blood collection.

As the oral route of administration is more convenient than the injection, patients will always preferably be switched from parenteral to oral anticoagulant therapy. DOACs are generally started after the discontinuation of heparins [35]. However, in some clinical settings, concomitant administration of LMWHs and DOACs may take place, especially when the temporary discontinuation of the DOACs may increase the risk of a thromboembolic event [36], or when the elimination of heparins is extended, e.g., in chronic kidney disease [37]. Thus, we simulated the clinical scenario when enoxaparin and DOACs are both present in the blood samples and found elevated results of the TT test and anti-factor Xa activity when compared with samples with a DOAC alone. BIOPHEN™ DiXaI, anti-factor Xa activity kit, was previously used to measure the activity of apixaban, edoxaban, or rivaroxaban in human plasma in the presence of enoxaparin, although the measured values seem to be a bit higher in the control groups, when compared with assays without a heparin inhibitor [9]. We also showed efficient neutralization of enoxaparin by polybrene or HBC, which enabled precise monitoring of rivaroxaban activity. Additionally, we demonstrated that both heparin inhibitors added to the sample containing enoxaparin before performing the commercial assay can also be successfully used for the monitoring of dabigatran activity in the TT test. When comparing HBC and polybrene, it should be noted that HBC restored anti-factor Xa activity to the levels of samples incubated with rivaroxaban alone in the limited range of heparins’ concentration. The weaker inactivation of heparins by HBC is probably because we used its lower than polybrene concentration, which was neutral in the anti-factor Xa assay. Depending on the heparin dosage, the peak of anti-factor Xa activity may reach 1.0 IU/mL; nonetheless, the anti-factor Xa activity measured a few hours from the injection of enoxaparin does not exceed 0.5 IU/mL, a value corresponding to the concentration of 5 µg/mL in our experimental settings [38,39]. Thus, the addition of HBC at a concentration of 10 µg/mL to blood samples should still be sufficient to completely inhibit any residual enoxaparin, thus enabling the estimation of the anticoagulation from rivaroxaban itself. Slight differences in the control and DOACs groups resulted from performing experiments with UFH and enoxaparin independently.

Our study has some limitations. Only a single concentration of dabigatran and rivaroxaban was assessed. It fits the lowest concentration considered important for presurgical interventions. However, patients on DOAC therapy are likely to have higher levels than that. We assessed only dabigatran and rivaroxaban as two representative DOACs. It would be expected that other DOACs (e.g., apixaban, edoxaban) would behave likewise owing to their similar mechanism of action to rivaroxaban.

## 5. Conclusions

We found that HBC and polybrene effectively neutralize the anticoagulant effect of heparins in human plasma in two coagulation tests: TT and anti-factor Xa activity. We have clearly shown that the use of both compounds eliminates the result of contamination of blood samples with heparins enabling accurate monitoring of dabigatran or rivaroxaban efficacy. It is necessary to expand our studies to develop standard protocols for the combinations of particular coagulation tests and the anticoagulation agent.

## Data Availability

The data presented in this study are available on request from the corresponding authors.

## Supplementary Materials

The following supporting information can be downloaded at https://www.mdpi.com/article/10.3390/jcm11082236/s1, Table S1: Characteristics of healthy volunteers; Figure S1: Inhibition of UFH at concentration 0.25; Figure S2: Inhibition of enoxaparin at a concentration of 2.5; Figure S3: Inhibition of UFH at concentration 0.25; Figure S4: Inhibition of enoxaparin at concentration 2.5.
