# Supplementary material for: Monitoring of Anticoagulant Activity of Dabigatran and Rivaroxaban in the Presence of Heparins
Source: J Clin Med. 2022 Apr 16;11(8):2236. doi: 10.3390/jcm11082236 (PMC9028841; doi:10.3390/jcm11082236)
Supplement: Supplementary file 1 [file jcm-11-02236-s001.zip › jcm-1627624-supplementary.pdf]

Supplementary Materials

Table S1. Characteristics of healthy volunteers.

	**Values in Healthy Patients**
Sex (M/F)	4/4
Age (years)	31 ± 6
BMI (kg/m^2)	23.4 ± 2.0
White blood cells (×10^3 μL)	4.7 ± 1.3
Red blood cells (×10^6 μL)	4.9 ± 0.2
Hemoglobin (g/dL)	14.6 ± 1.0
Hematocrit (%)	43.0 ± 2.5
Platelet (x10^3 μL)	238.1 ± 48.8
Creatinine (mg/dL)	0.85 ± 0.15
Total cholesterol (mg/dL)	172.9 ± 35.4
Triglycerides (mg/dL)	71.8 ± 20.6
HDL (mg/dL)	53.7 ± 10.4
LDL (mg/dL)	106.4 ± 32.0
Total bilirubin (mg/dL)	0.82 ± 0.31
ALT (U/L)	28.4 ± 26.2
CRP (mg/L)	1.5 ± 2.6
Blood glucose level (mg/dL)	93.9 ± 5.8
SBP (mmHg)	120.1 ± 4.1
DBP (mmHg)	77.0 ± 7.1

Data are shown as mean ± S.D. ALT, alanine transaminase; BMI, body mass index; CRP, C-reactive protein; DBP, diastolic blood pressure; HDL, high-density lipoprotein; LDL, low-density lipoprotein; SBP, systolic blood pressure.

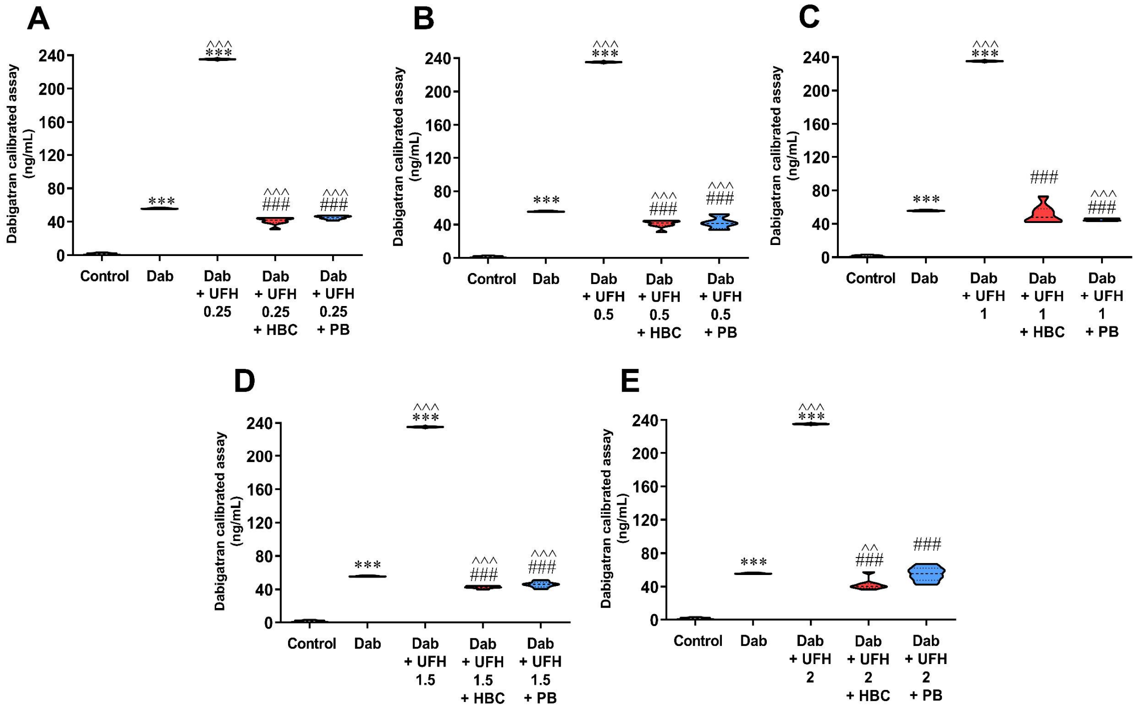

Figure S1. Inhibition of UFH at concentration 0.25 (A), 0.5 (B), 1 (C), 1.5 (D) and 2 U/mL (E) by HBC or polybrene at a concentration of 50 µg/mL during testing of dabigatran (50 ng/mL) anticoagulant activity by TT converted to ng/mL for dabigatran in human plasma. ***$p < 0.001$ vs control; ^^$p < 0.01$, ^^^$p < 0.001$ vs dabigatran; ###$p < 0.001$ vs dabigatran with UFH, Mann-Whitney test. Results are shown as violin plots with minimum and maximum values, and analyzed with GraphPad Prism 8 software. Dab, dabigatran; HBC, heparin-binding copolymer; PB, polybrene; UFH; unfractionated heparin.

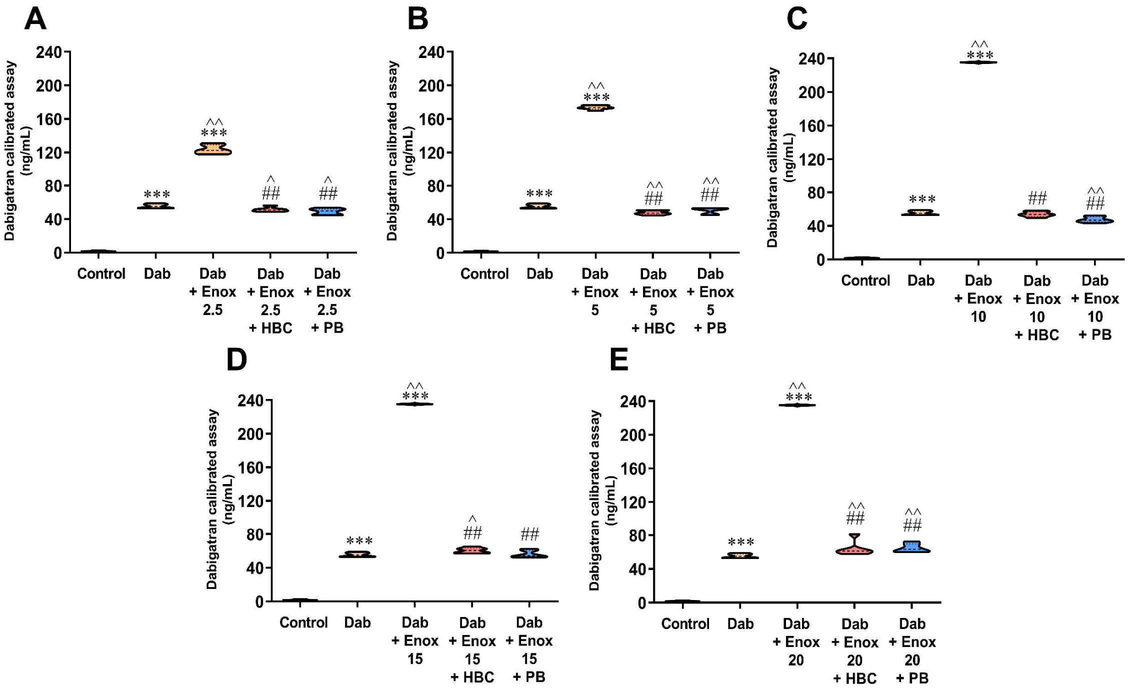

Figure S2. Inhibition of enoxaparin at a concentration of 2.5 (A), 5 (B), 10 (C), 15 (D) and 20 μg/mL (E) by HBC or polybrene at a concentration of 50 μg/mL during testing of dabigatran (50 ng/mL) anticoagulant activity by TT converted to ng/mL for dabigatran in human plasma. ***$p < 0.001$ vs control; ^$p < 0.05$, ^^$p < 0.01$ vs dabigatran; ##$p < 0.01$ vs dabigatran with enoxaparin, Mann-Whitney test. Results are shown as violin plots with minimum and maximum values, and analyzed with GraphPad Prism 8 software. Dab, dabigatran; Enox, enoxaparin; HBC, heparin-binding copolymer; PB, polybrene.

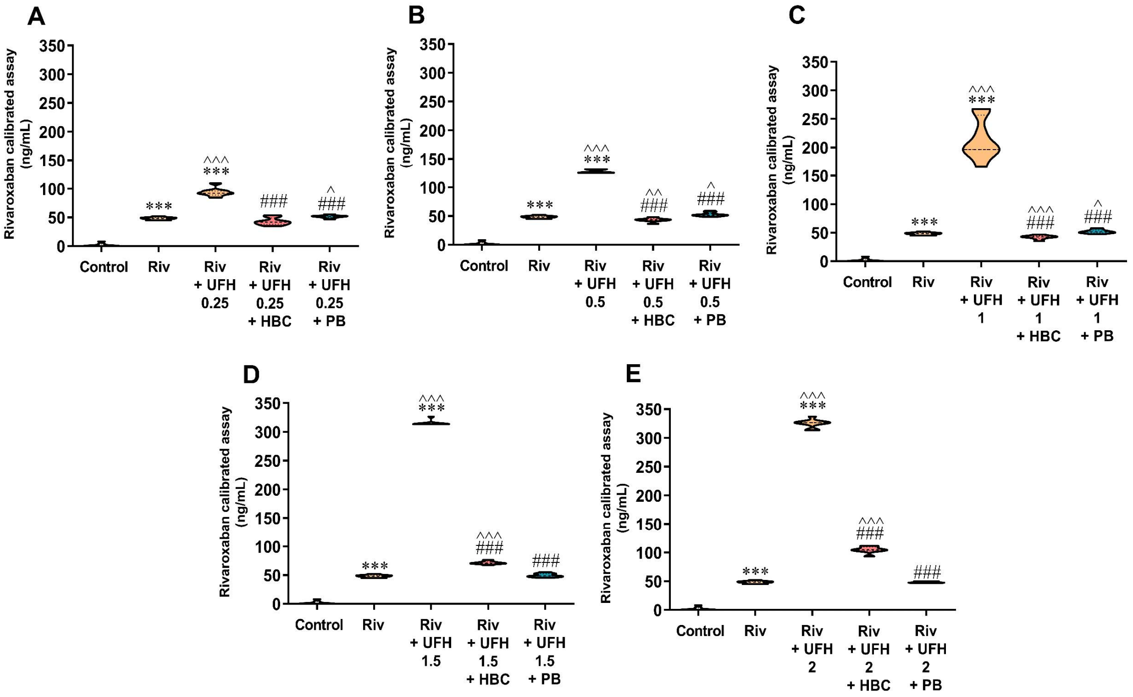

Figure S3. Inhibition of UFH at concentration 0.25 (A), 0.5 (B), 1 (C), 1.5 (D) and 2 U/mL (E) by HBC (10 µg/mL) and polybrene (50 µg/mL) during testing of rivaroxaban (50 ng/mL) anticoagulant activity converted to rivaroxaban calibrated anti-factor Xa assay in human plasma. ***$p < 0.001$ vs control; ^$p < 0.05$, ^^$p < 0.01$, ^^^$p < 0.001$ vs rivaroxaban; ###$p < 0.001$ vs rivaroxaban with UFH, Mann-Whitney test. Results are shown as violin plots with minimum and maximum values, and analyzed with GraphPad Prism 8 software. HBC, heparin-binding copolymer; PB, polybrene; Riv, rivaroxaban; UFH, unfractionated heparin.

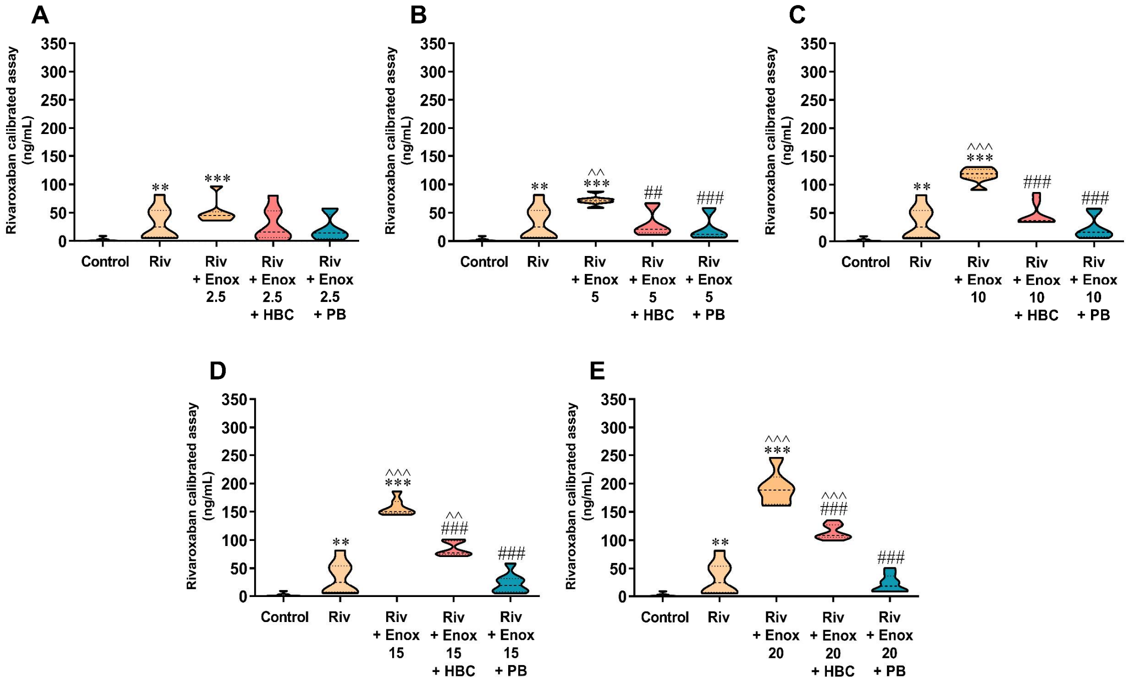

Figure S4. Inhibition of enoxaparin at concentration 2.5 (A), 5 (B), 10 (C), 15 (D) and 20 μg/mL (E) by HBC (10 μg/mL) and polybrene (50 μg/mL) during testing of rivaroxaban (50 ng/mL) anticoagulant activity converted to rivaroxaban calibrated anti-factor Xa assay in human plasma. **p < 0.01, ***p < 0.001 vs control; ^^p < 0.01, ^^^p < 0.001 vs rivaroxaban; ##p < 0.01, ###p < 0.001 vs rivaroxaban with enoxaparin, Mann-Whitney test. Results are shown as violin plots with minimum and maximum values, and analyzed with GraphPad Prism 8 software. Enox, enoxaparin; HBC, heparin-binding copolymer; PB, polybrene; Riv, rivaroxaban.
